# How do UK-based university students understand family support in relation to the impact it may have on their wellbeing?

**DOI:** 10.3389/fpsyg.2025.1393594

**Published:** 2025-12-16

**Authors:** Iona Adams, Aisha Toorawa, Danae Finiri, Chloe Schofield, Anamaria Churchman

**Affiliations:** Department of Psychology, Faculty of Biology, Medicine and Health, University of Manchester, Manchester, United Kingdom

**Keywords:** wellbeing, family support, students, parental support, university

## Abstract

**Introduction:**

A person’s wellbeing is influenced by many factors at various stages of life, and research has found that family support is hugely important in its maintenance. During adolescence, (as a result of many biological and cognitive changes which take place during this stage), many young people might experience decreased wellbeing. Such difficulties are further exacerbated in university students, with research showing that this age group are prone to experiencing high rates of mental health difficulties. Various quantitative studies have investigated student mental health, yet there has been little focus on the subjective experiences of wellbeing and social support. The current study aimed to gain a deeper understanding of how university students perceive family support and the impact this might have on their wellbeing.

**Methods:**

Due to the nature of the study, an advert was placed for all Psychology undergraduate students at a university in the North-West of England. A total of 16 semi-structured interviews were conducted. The interviews were analyzed using thematic analysis.

**Results:**

Three main themes were identified: “type of support,” “family context” and “balance and adjustment.”

**Discussion:**

The findings highlighted that university students view family support as vital in maintaining positive wellbeing, and that parental support was valued the most among various types of family support. This included both emotional and financial support. Unlike the wider literature, in the current study, some students considered financial support to be less necessary while others suggested that wellbeing may be negatively impacted by inadequate monetary support. Furthermore, family background such as culture and socio-economic background was seen to greatly influence the support provided. Lastly, students emphasized that family support can be beneficial when a balance is stricken, and support is neither overbearing nor lacking. Students highlighted how divorce or absent parents can negatively impact students’ wellbeing. Similarly, helicopter parenting or parents who sought to control and put pressure were perceived as overbearing. Offering the right support that met the needs of students at the right time was classed as adequate.

## 1 Introduction

### 1.1 Wellbeing

Wellbeing can be impacted by many factors throughout life and has been described as more than simply the absence of illness (Deiner et al., 2009). van Agteren et al. (2021) define wellbeing in terms of varying subjective and psychological aspects, such as overall life satisfaction and the influence of positive relationships in one’s life. The most holistic definitions of wellbeing recognize that part of being human involves experiencing both highs and lows, and this has been called “sustainable wellbeing” (Huppert and Cooper, 2014). Sustainable wellbeing is rooted in the goal of functioning well (as opposed to feeling happy all the time) which Huppert and Cooper (2014) suggests is only possible if the full range of emotions and experiences are acknowledged.

One significant factor in the maintenance of wellbeing is social support, which was identified by Pinkerton and Dolan (2007) as being crucial for successful coping. Whittaker and Garbarino (1983) describe social support as the “bread and butter” source of help. Social support is thought to be accessed primarily from family members, although research shows that other sources include friends or significant others (Hefner and Eisenberg, 2009). This support has been described by Turner and Brown (2010) as “multidimensional,” with many interconnecting elements. Emotional aid in the form of empathy, listening and generally “being there” for someone is considered to be one of the most fundamental forms of support a family can provide (Cutrona, 2000; Pinkerton and Dolan, 2007). Research suggests that one of the most important aspects of emotional support in the maintenance of wellbeing is talking and communication (Cooper, 2013). This is prevalent also in family contexts, where support from parents has been revealed as crucial to mental health and development (Turner and Brown, 2010). The quality and type of family support received has been shown to impact upon the success of any coping strategies used (Lohman and Jarvis, 2000).

### 1.2 Family support

The impact of family support on mental health and wellbeing has been studied extensively with many groups of individuals. Results converge to show that mental health outcomes are closely linked to the amount of family support received (McConnell et al., 2016; Wallace et al., 2016). It has been found that, following traumatic events, lower rates of depression were experienced by individuals who received ample family support (Lakey and Cronin, 2008; Suwinyattichaiporn and Johnson, 2022). A significant aspect of family support is the parent-child relationship. Attributed as one of the most important parts of life, research has revealed that the quality of this relationship in childhood is closely linked to wellbeing later in life (Smart et al., 2008). Armsden and Greenberg (1987) researched aspects of life they believed to be integral to wellbeing (such as response to stressful life events) and found that high quality parent-child relationships had a significant positive impact on these. In support of these findings, research by Bruffaerts et al. (2018) also found that children with a high level of connectedness to their parents had increased self-esteem, along with social competency. Both of these “internal assets” contribute to an individual’s healthy development, and therefore their overall state of wellbeing. This indicates that parent-child relationships are fundamental in the maintenance of wellbeing.

### 1.3 Adolescence

Family support is hugely beneficial for wellbeing throughout life, but this support becomes particularly crucial during adolescence (van Harmelen et al., 2016). From the ages of 10 to 19 years old, young people go through a time of immense change and growth, the pace of which has been compared to that of infancy (UNICEF, 2002). During this time, young people experience many changes such as cognitive maturation and the onset of puberty (Sawyer et al., 2018). However, along with these biological developments, adolescence also sees a social and emotional revolution (UNICEF, 2002). Throughout the teenage years, young people become increasingly responsible and seek higher levels of independence, alongside a wealth of other developments (Sawyer et al., 2018). Adolescents are often forced to deal with these changes alongside stresses such as academic achievement and friendship struggles (Compas, 1987). This combination consequently results in adolescence being a challenging time for individuals, and one that could inevitably lead to anxieties and an overall reduction in wellbeing (UNICEF, 2002). Adolescence, consequently, has been described as the “foundation for future health” (Sawyer et al., 2012, p. 1631).

### 1.4 Students

The stresses experienced during adolescence are often intensified for another group: university students. In addition to the challenges outlined above, students must navigate significant transitions such as moving away from home and establishing new support networks. In the UK, the proportion of students entering university at age 18 rather than later in life has risen in recent years (Universities UK, 2019). Consequently, more students than ever are beginning university during late adolescence—a factor that places them at heightened risk of mental health difficulties (Wadman et al., 2019). For most individuals, the university years mark a transition from late adolescence to early adulthood (Bruffaerts et al., 2018), a period psychologists describe as “developmentally crucial” (Arnett, 2000). Evidence suggests that wellbeing often declines during this stage (Macaskill, 2012). A review by Storrie et al. (2010) found that, globally, 83% of students reported being moderately or severely distressed. Furthermore, university students are more likely to experience psychological distress than their non-student peers (Cvetkovski et al., 2012), highlighting the vulnerability of this population. Despite these challenges, students do not face this transition alone. Research indicates that support networks—primarily family and friends—serve as the main resource for coping with stress and change (Hombrados-Mendieta et al., 2012). During this developmental stage, reliance on these networks is particularly high, more so than at any other point in life (van Harmelen et al., 2016).

### 1.5 Family support and wellbeing

Family support can take multiple forms and often varies across individuals and contexts. The literature identifies two primary types of family support: emotional and instrumental (Leung et al., 2020). Emotional support, sometimes described as intangible, refers to care expressed through encouragement, empathy, and understanding. In contrast, instrumental—or tangible—support involves practical assistance, such as help with household tasks or financial support (Leung et al., 2020). However, examining family support requires more than simply acknowledging its presence. Lohman and Jarvis (2000) argue that wellbeing cannot be fully understood without considering the broader family context, as its maintenance is inherently context dependent. One critical contextual factor is socioeconomic status (SES). Research consistently demonstrates a strong link between SES and mental health, with economic deprivation associated with increased risk of psychological difficulties (Reiss, 2013). In a large-scale systematic review, Reiss (2013) found that children from socioeconomically disadvantaged families were two to three times more likely to experience mental health problems compared to their more privileged peers. This influence extends to students, where low SES is frequently cited as a barrier to both academic achievement and stable wellbeing (Devlin and McKay, 2011; Hefner and Eisenberg, 2009). Limited financial resources may constrain the provision of instrumental support, further compounding these challenges. Beyond economic factors, a person’s cultural background also shapes family support and wellbeing. Cultural norms strongly influence parenting styles, particularly in educational contexts (Yamamoto and Holloway, 2010). Parental attitudes toward academic success—and the strategies employed to achieve it—are deeply rooted in cultural values. Yamamoto and Holloway (2010) suggest that these differences arise from the skills and characteristics prioritized within a given society, which in turn inform parenting practices and expectations. In sum, family support is multifaceted, encompassing emotional and practical dimensions while being shaped by socioeconomic and cultural contexts. Understanding these interconnections is essential for promoting wellbeing across diverse populations.

Research into factors that can reduce the quality of family support is extensive and has revealed that one of the main risk factors is parent separation. Kroese et al. (2020) found that the consequences of growing up with divorced parents are huge. Children raised by divorced parents are more likely to become anxious and to be involved in criminal activity. Furthermore, research suggests that children within single-parent families may experience an increased number of stressors, arguably leading to a decrease in support quality. Another factor with the potential to reduce the quality of support is parenting style. In comparison to authoritarian parenting styles, authoritative parenting has been associated with reduced behavioral difficulties and better emotion regulation in children, regardless of cultural background (Haslam et al., 2020). Research has revealed that the ways individuals cope with stress impacts wellbeing to a greater extent than the stressful events themselves (Lohman and Jarvis, 2000). Coping processes play a critical role in shaping the impact of family support on wellbeing. While family support provides resources—both emotional and instrumental—that can buffer stress, the effectiveness of this support often depends on how individuals interpret and utilize it through their coping strategies. Coping can act as a mediator by explaining how family support translates into improved wellbeing; for instance, supportive families may encourage adaptive coping behaviors such as problem-solving or seeking help, which in turn reduce stress and enhance psychological wellbeing. Conversely, coping can also function as a moderator, influencing the strength of the relationship between family support and wellbeing. Individuals who rely on maladaptive coping strategies, such as avoidance or rumination, may derive limited benefit from family support, whereas those employing adaptive strategies experience greater positive effects. Thus, coping processes are not merely reactive mechanisms but pivotal factors that determine whether family support fosters resilience or fails to mitigate stress (Lohman and Jarvis, 2000).

### 1.6 The present study

Although the literature surrounding the topic is extensive, qualitative research into the influence of family support on student wellbeing is currently sparse. Previous studies have almost exclusively employed quantitative methods when researching wellbeing, creating an objective view of the construct. This limits the extent to which the unique experiences of the demographic of interest can be fully understood. Arguably it neglects the level of nuance required by such a subjective topic (van Agteren et al., 2021). The present study extends the literature surrounding the influence of family support on wellbeing. On the basis that quantitative methods bring certain limitations, qualitative methods are used to expand on and complement existing knowledge by exploring what university students understand of the topic. Previous studies have employed surveys and questionnaires to demonstrate links between family support and wellbeing, but a more in-depth analysis and understanding of the topic is still outstanding.

The current research therefore aims to expand on the quantitative literature available and provide a qualitative view of the topic. In addition, much of the research into mental health and wellbeing has been conducted on younger adolescents due to the period of intense change they enter in their teen years (UNICEF, 2002). The university years are a huge transitional period for young adults. Therefore, the current study aims to explore what students think about the role of family support and its link to wellbeing.

Another gap in the literature pertains to the locations in which previous studies or meta-analyses have been carried out. There exists a considerable body of literature on the impact of family support for individuals in the US and elsewhere (Cvetkovski et al., 2012; Hefner and Eisenberg, 2009) However, there is a distinct lack of focus on UK-based university students. This research therefore addresses the underrepresentation of UK-based students in the existing literature on family support and wellbeing.

Through the use of semi-structured interviews, the current study seeks to provide a more in-depth view of students’ unique perspectives of the topic.

## 2 Materials and methods

This is a qualitative study, in which semi-structured interviews were used to collect data. Ethical approval (reference 2023-15824-26758) was obtained from the institution where the study was registered prior to recruitment taking place. Each participant was provided with an information sheet detailing what their participation would require. Participants gave written consent prior to being interviewed and were informed of their right to withdraw their data at any time, up to seven days after completion of the interview. Further verbal consent was obtained prior to recordings commencing. Potentially identifiable information has been excluded from this report.

### 2.1 Design

Previous studies of family support and wellbeing have not focused on unique, subjective experiences due to the quantitative methods often used. The current qualitative approach allowed for specific questions surrounding students’ opinions of wellbeing, whilst also enabling participants to steer the conversation. A total of 16 interviews were recorded and transcribed verbatim by four researchers. Responses were analyzed using thematic analysis, with Braun and Clarke’s (2006, 2014) guide supporting the process. Thematic analysis was chosen due to the thorough, step-by-step process defined by Braun and Clarke (2006, 2014), which allowed for consistency among the researchers. Themes were identified using an inductive approach, and coding was carried out by all four researchers. One of the main benefits of thematic analysis for this research is its flexibility, meaning that the data obtained did not need to fit a particular narrative or pattern (Braun and Clarke, 2020). This was important as the current study was interested in examining the opinions held by students, and researchers aimed to remain flexible in the topics and conversations that arose during the interviews. Thematic analysis is particularly useful for researching subjective phenomena such as wellbeing and therefore aided in providing a more nuanced exploration of the topic than previous studies. The aim of this study was to explore participants’ experience of family support and wellbeing at university in a rich and detailed way, aiming to move beyond purely observational findings. Thematic analysis is also recommended for use in health and wellbeing research (Braun and Clarke, 2014). These various merits were considered when selecting it for use in this context.

The current study assumes the existence of an objective external reality whilst acknowledging that the experiences being explored are subjective. The epistemological perspective most aligned with the current study is therefore critical realism (Fletcher, 2017).

### 2.2 Participants

A total of 16 first and second-year psychology students from a university in the North West of England were recruited to take part in the study. Opportunity sampling allowed researchers to access the specific demographic of interest, and available individuals were able to register as such. All participants were recruited through online advertising, with the study being internally advertised through the University’s system. Participants received compensation in the form of credits, with one credit awarded for every 15 min of their time. The online advertisement described the study, and the contact details of each researcher were available for anyone who sought more information.

Interviews were conducted via Zoom, and therefore it was necessary that participants had adequate hearing capabilities to communicate with the researcher. All were fluent in both understanding and speaking the English language. None of the participants were currently experiencing mental health difficulties or seeking support. The interviewees were comprised of 15 females and one male. The mean age recorded was 18.81 years. Individuals from a range of ethnicities took part, and full details are available in Appendix A.

### 2.3 Data collection

Following an in-depth review of the literature, a topic guide was developed which outlined key themes to be covered in the interviews. The guide included topics such as students’ understanding of family support, and how family background may impact the support provided. Broad, open-ended questions were generated on the basis that they could provide novel findings regarding the research aims and target topics. The topic guide provided a loose interview structure whilst allowing for flexibility and natural flow of conversation, and is available in Appendix B.

Participants completed a demographics form on Qualtrics designed to obtain data such as age, gender and ethnicity, and could decline to provide an answer if they wished.

### 2.4 Analysis

All researchers took part in training designed to highlight the importance of being inquisitive and keeping all follow-up interview questions open-ended. It is important to acknowledge individual differences in perspectives when interpreting meaning from the data collected, particularly in qualitative research (Warren, 2001). Braun and Clarke (2020) highlight that the coding process in thematic analysis is entirely subjective due to differences in perspectives and assert that coding therefore requires reflexive researchers. In the current study, researchers remained aware of the influence of their own subjective experiences when generating codes, themes and ultimately interpreting meaning. Differences in perspectives could be a result of many things, including an individual’s race, class or sexuality. Meaning can be derived not only from the participant’s perspective but also from the researcher’s (Warren, 2001), and this was acknowledged during all stages of interviews and analysis.

Thematic analysis was advantageous for the current study as it facilitated a focus on the subjective experiences of the participants whilst providing a systematic method of assessing a large body of data. Due to the nature of this analysis style, along with all researchers being students and therefore belonging to the demographic of interest at the time, there is a chance of potential bias in the analysis. Although researchers strived to remain objective, all had varying experiences of both family support and wellbeing during their time at university which inevitably will have impacted the analysis and interpretation of findings. To mitigate the impact of these potential biases, researchers were considerate of their own influence upon the research process and remained reflexive and aware throughout.

The interviews were recorded via zoom and transcribed by the interviewers. The transcripts were then combined and read thoroughly by all researchers to ensure familiarization of the data set. Then, codes were formed based on the content of the transcripts. At this point, the researchers met to discuss the codes that had been identified, going through all transcripts together and confirming that the whole data set was represented. The codes included topics relating to support across the lifetime and factors that can impact the support provided, among others. These were sorted into groups of related codes which resulted in the formation of initial themes. These initial themes were then organized into overarching groups of similar themes which were reviewed to check for consistency. Three overarching themes were identified at this point: “type of support,” “family context” and “balance and adjustment,” each of which contained relevant subthemes. Researchers then returned to the data set and repeated the coding process again to ensure that all codes were included within the selected themes, and the whole data set was accounted for.

## 3 Results

**FIGURE 1:**
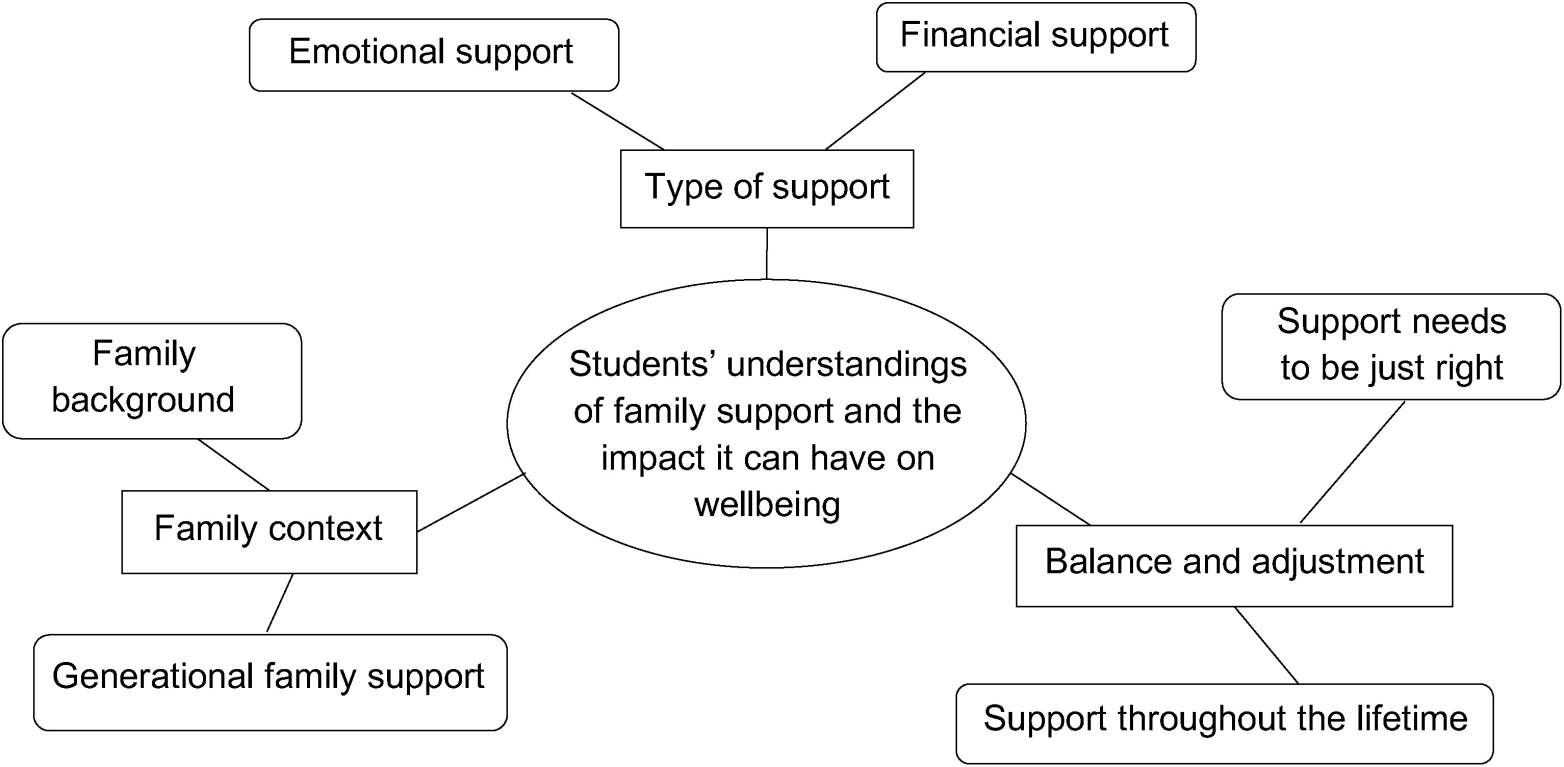
A Thematic map of the themes and subthemes identified during analysis.

All 16 participants shared the view that family support was vital for maintaining wellbeing. The students drew upon their own experiences and the experiences of those around them when describing their perceptions of how support can influence wellbeing. As such, three main themes were identified: “type of support,” “family context,” and “balance and adjustment.” Each of these themes contained further subthemes, discussed below. A thematic map of the findings is presented in Figure 1. A table detailing how many participants mentioned each subtheme as well as how often the subthemes were directly linked to wellbeing has also been included below (Table 1).

**TABLE 1:** Frequency of subthemes and links to wellbeing.

Sub-theme	How many participants mentioned it	Explicit links between subtheme and wellbeing
Emotional support	All	17
Financial support	All but one	40
Family background	All	63
Generational family support	Majority (11)	6
Support needs to be just right	All but one	22
Support throughout the lifetime	All	17

### 3.1 Type of support

This theme captures participants’ views on types of support and how these can be beneficial for wellbeing. Participants valued the process of talking through their problems and also acknowledged how financial support was crucial for healthy development. The theme contains two subthemes: “emotional support” and “financial support.”

#### 3.1.1 Emotional support

Participants described how emotional support often comes in the form of talking and communication with loved ones, specifying that this is a crucial aspect of the support provided by the family. Participants noted that not having anyone to talk to can lead to feelings being “bottled up” (participant 5), and it was frequently expressed that this can be detrimental to wellbeing: “not having someone to be at home asking “How are you doing this week? Are you okay?” really might, like, negatively impact because then you’re holding everything in and you’ve not got anyone to talk to” (participant 31). A strong recurring idea was that parents act as an unofficial therapist to whom individuals can verbalize their difficulties, and this was seen as essential for wellbeing. Participants described feeling relief after calling their parents: “every time when I felt like “I can’t do it anymore,” I just call my family, video call or text them…I feel much better after texting them, after talking to them” (participant 4). It was expressed that in order to be beneficial for wellbeing, emotional support from parents must be unconditional: “viewing love as conditional can definitely reduce the, the support because, well, the quality of the support, because it kind of just feels artificial in that sense like, it’s not meaningful, it’s not genuine” (participant 5).

#### 3.1.2 Financial support

Along with emotional support, participants reported that financial support was integral to the maintenance of wellbeing. Several participants described how financial support is crucial for healthy development, particularly during childhood and adolescence when there is increased dependence on caregivers: “we can’t really get a job at 8 years old, so we do need financial support, like, to be fed, like, to have a warm house” (participant 1). One participant also explained how practical help from families may act as a buffer from stress in difficult times. They mentioned that students lacking financial support from loved ones may “struggle” if they “don’t have anything to fall back on” (participant 11). Although participants viewed financial support as beneficial for wellbeing, lack of financial support was not seen as inherently detrimental. Conversely, many participants expressed greater desire for emotional support: “I would much rather my parents be there to support me when I’m emotionally low rather than struggling financially” (participant 32).

### 3.2 Family context

This theme is comprised of two subthemes concerning the contextual factors that can affect the support a family provides. Participants emphasized how certain types of support might be more important than others, and that this “just depends on like individual differences” (participant 24). Participants asserted that a family’s context, such as their culture and history, would be influential in determining the style of support provided. This theme contains two subthemes relating to the idea that support is not “one-size-fits-all”: “family background” and “generational family support.”

#### 3.2.1 Family background

Cultural background was seen as a key explanation for the differences in support that families provide. Participants reported that culture can affect people’s wellbeing to different extents. One student detailed how cultural acceptance of individual differences such as sexuality may impact support: “some families are, like, accepting of people like of the LGBTQ, but, other, like, for example, if my parents are like Pakistani, or like from a South Asian background, they would be less acceptive of that. So I feel like it’s, like it’s everyone’s own cultural and religious views” (participant 2). Participants also described how family structures and dynamics vary across the globe, with priorities differing between cultures: “I think, some countries are more family orientated than probably a Western British one. I think, like the extended family in more third world country seems more important” (participant 31). Participants expressed an awareness of the fact that culture influences the expectations parents place upon their children, and therefore the support they provide: “I mean cultures parent differently. They have different approaches to what they think is best for their children so I think that probably impacts how they support them” (participant 12). The same participant went on to describe how a family’s socioeconomic status can heighten the stress an individual is exposed to, potentially threatening wellbeing: “I think it probably impacts your mental health as well, as if you don’t have the funds, for like your life, and then your family may not have the funds to help you. That definitely makes, can make you stressed” (participant 12). All participants were aware that, in a practical sense, a family’s economic status would directly relate to the types of support they are able to provide: “then obviously, people’s socioeconomic background is gonna have an influence on what actually can physically be provided for” (participant 33).

#### 3.2.2 Generational family support

Participants noted that individuals model behaviors from those around them, and that many parents raise their children similarly to how they themselves were brought up: “people really, uh, imitate, um, other’s behavior, so, um, how, maybe, how grandparents taught our parents are also, um, transferred to how our parents taught us” (participant 3). Students regularly referred to how this process of modeling support from others can result in support styles being passed down through generations: “the support given to your family like to you by your family depends on how your family kind of got like support from their parents, and then it’s like this whole kind of cycle” (participant 22). Some participants reported that the role models present during a person’s younger years are extremely influential for their future wellbeing: “individuals are] more likely to be able to look after their self if they have sort of been modeled how to” (participant 32). Additionally, participants reported the support system during childhood to be “massively impactful on who you are as an adult” (participant 12). It was clear that participants viewed positive parent-child relationships as beneficial to the wellbeing of future generations: “our parents’ upbringing if they had that relationship with their parents where they were supported and felt supported, I think it’s easier for them to do the same with their own children” (participant 21).

### 3.3 Balance and adjustment

A central view that participants held was that family support should adapt to individual needs throughout different stages of life. Many participants referred to the huge number of changes humans experience throughout their lifetime, stating that the support provided should adjust depending on these. Participants also emphasized the importance of reaching a balance between too little and too much support. This theme contains two subthemes: “support throughout the lifetime” and “support needs to be just right.”

#### 3.3.1 Support throughout the lifetime

Participants recognized that the amount of support required will change throughout different stages of life, but acknowledged the necessity of it remaining available. Participants stressed that, even during times when individuals are less dependent on their parents, support should still be accessible: “although I’m far away, I’m living my own life, I am an adult, that support is still somehow there” (participant 2). Many participants described how it is natural for increased independence to result in distance from the family, along with less reliance on them and an expansion of the support network: “as you get older, you meet more people, and you form your own kind of support network of your own, you know. That’s not the one that you’ve just kind of been given” (participant 33). Not only does the amount of support change over time, but a few participants mentioned how the types of support may shift from practical to more advice-seeking: “as a child, you probably get more like a lot more support… like helping with friends and schoolwork and things. And yeah, just parenting in general but as you get older it’s more having someone to talk to, and ask for advice and different aspects of life and money” (participant 12). Many participants also emphasized the importance of support being available even in the absence of negative life events, and regardless of the stage of life someone is in: “you don’t even have to be distressed you could just like go up to them, and they’ll provide you with like, you know, whatever kind of support you need” (participant 22).

#### 3.3.2 Support needs to be just right

Participants reported that not receiving enough family support may lead to individuals feeling “isolated” (participant 5) and “lost” (participant 1) and can result in “feelings of hopelessness” (participant 21). Participants described how inadequate family support can cause stress later in life, stating that “someone who did not have such good family support, might like face some amounts of distress” (participant 22). Some participants were unclear as to why this distress could occur, whilst others attributed it to the fact that the individual may look for support from unhelpful sources: “I feel like they will tend to seek for support from other people like their friends, and not from their family. And I’d say, if they make friends with people who are, who brings, who brings them bad impact, yeah, that could be dangerous” (participant 4). Participants were also acutely aware of the impact that life-changing events can have on the family unit, and its potential to reduce support quality: “If there was a death that was very close to home… or maybe like a divorce… that could really impact support, I think cause sometimes when parent’s divorce like it becomes very, um, like nasty like that, it’s very negative and I think that can really impact how a child is supported” (participant 5).

In addition, participants often described how over-involved or controlling parents can cause their children stress. It was clear that participants believed support could be useful only to a point, and that excessive support could become controlling, toxic and negative: “parents should know, like, what is the boundary between you and your children” (participant 35). Participants also emphasized the importance of young people being allowed to make mistakes and learn from them, arguing that parents should steer their children as opposed to forcing them in certain directions: “adults and parents’ jobs, I think, is to lead them guide them, but not just say “you have to do that because I’m an adult” “(participant 34). Additionally, multiple participants alluded to the adverse effects that can result from individuals being put under too much pressure from their parents: “some families push their kids way too far. And especially at university, you can see some kids are just living under their parents’ shadows” (participant 1). The same participant went on to describe how taking a gentler approach could make parental support more beneficial: “they provide support for your goals, but they don’t, like, place their own, you know, they don’t live through you. They want you to do what you want to do.”

Overall, the 16 interviews revealed clear patterns demonstrating how family support can directly or indirectly influence wellbeing. Emotional support was described as overwhelmingly positive, consistently labeled as essential for mental health, coping, and confidence. Financial support was perceived as having mixed effects—beneficial when it provided and enhanced opportunities but detrimental when it was absent or overemphasized. Family background and generational family support also generated mixed feedback from participants; according to participants cultural norms and multi-generational households can often foster closeness but they can also create pressure or feel intrusive. The subtheme “support needs to be just right” highlighted negative impacts when support was excessive or controlling, while “support throughout the lifetime” was largely positive, though its importance diminishes in adulthood and withdrawal after strong dependence can harm wellbeing. Overall, emotional support was the strongest protective factor, while imbalance—whether too much or too little—was a recurring risk across themes.

## 4 Discussion

The current study aimed to explore students’ understandings of family support and its impact on wellbeing. University students reported that family support was a crucial aspect in the maintenance of wellbeing. Participants demonstrated an awareness of multiple types of support provided by the family, such as financial and emotional. The latter was particularly valued by participants, who often referred to the importance of sharing problems with their parents. Students recognized that a family’s context can have huge influence on the support an individual receives, and that it is necessary to consider factors such as socioeconomic status and cultural background when examining wellbeing. Additionally, participants emphasized that people may need different amounts and types of support depending on their stage of life or personal circumstances and stressed the importance of support being tailored to the individual.

Although research has consistently highlighted the importance of social support for wellbeing, previous studies have generally relied on quantitative methods. The current study employed semi-structured interviews, representing one of the very few qualitative studies on this topic. The present study adds to the existing literature on family support, incorporating participants’ voices into the exploration of its impact on wellbeing. Some similarities with previous research were found, such as themes surrounding the importance of emotional support (Pinkerton and Dolan, 2007) and how individuals require varying levels of support at different stages of life (Compas, 1987). Themes less prevalent in the literature were also identified. These included the idea that parental support styles can be passed down to generations, a topic which has only recently appeared in research (Abraham et al., 2021).

Results indicate that emotional support in the form of open talking and communication was regarded as one of the most vital aspects of support parents can provide. Participants referenced that communication with loved ones can buffer the effects of loneliness, and many emphasized the role this plays in reducing isolation and thus improving wellbeing. Research shows that, particularly in university and college students, social support is key in reducing loneliness (Chang et al., 2017). Moreover, family support was shown to mitigate the risk of loneliness leading to suicide for these individuals (Chang et al., 2017). The current study therefore expands upon previous findings which have shown the positive therapeutic impact of talking (Cooper, 2013). Participants viewed talking as helpful as it allowed them to explore their problems with someone else. This appeared to remain beneficial even when the problems were simply talked through, which suggests that perhaps the benefits of talking are not rooted in problem solving but problem sharing. Furthermore, the current study found that communication with parents was almost therapeutic in nature, with the effects on wellbeing enhanced by parents demonstrating unconditional support. In a review of high school-based counseling techniques, Cooper (2013) found that talking was one of the most helpful parts of therapy for the students. Similar findings were shown in the current study, with talking being identified as vital for helping students work through their issues. This suggests that reciprocal conversation with others can help maintain wellbeing both in personal and clinical settings.

Almost all participants viewed parental support as the most vital aspect of support for their wellbeing, while support from the wider family was seen as less essential. Support from friends and other peers was perceived as less useful than support from parents, and participants referenced more advice-seeking behaviors in relation to their parents. This is consistent with previous findings that the parent-child relationship is hugely important and closely linked with wellbeing, even later on in life (Smart et al., 2008). These findings support the view that individuals rely on their parents not only for practical help, but also pastoral (Pinkerton and Dolan, 2007). Alternative support should be available for those individuals who may not have high quality relationships with their parents, as literature would suggest this poses a risk to their wellbeing. This support could perhaps be targeted at high school students during the start of their adolescence, where it is clear that parent-child relationships are vital.

Along with emotional support, research has shown financial provision to be a major component of the support a family provides (Leung et al., 2020; Pinkerton and Dolan, 2007). In the current study, participants viewed financial support as important for development, particularly when dependence on caregivers is high (such as during childhood). Provision of necessities such as food and shelter was mentioned as an essential aspect of financial support. This is in line with current literature which states that family support has been described as “multidimensional,” serving many purposes in an individual’s life (Turner and Brown, 2010). Despite acknowledging the importance of receiving multiple types of support, participants considered financial support to be less necessary. Some students reported that wellbeing may be negatively impacted by inadequate monetary support, but also that with good emotional support, these negative effects could be counteracted.

Consistent with related research, participants viewed cultural background as a factor which could impact family support. The type and quality of support were both mentioned as being influenced by culture, as were the individual differences a family accepts, such as sexual orientation. The family environment is closely related to mental health, and as such it has previously been argued that wellbeing cannot be considered without exploring an individual’s context, of which culture forms a huge part (Lohman and Jarvis, 2000). In the current study, participants highlighted that expectations on children differ between cultures. Research has consistently revealed evidence for cultural differences in child rearing practices, which could perhaps be explained by these differences in expectation. Studies have found variations in academic ambition between cultures, and research shows that there are cultural differences in the goals parents have for their children (Yamamoto and Holloway, 2010). Yamamoto and Holloway (2010) argue that this arises due to cultural differences in the skills or characteristics that are valued by society, which therefore impact the parenting styles that are employed. This supports the view that cultural background can influence whether parents employ strict or controlling support styles, and this is reflected in the current study. Although it is clear that culture influences family support, qualitative research into its effects on wellbeing is sparse. Future studies could focus on the reasons support styles vary across the globe and look into how to maintain wellbeing across all cultures. It is, however, imperative that this research honors cultural differences and does not impose Western ideals on parenting styles or ideas of wellbeing (Lambert et al., 2020).

Another aspect of family background with the potential to impact support was socioeconomic status. With reference to adolescence and the university experience, participants emphasized the advantages that can come from belonging to a family of higher socioeconomic status. Students believed that individuals with higher-earning parents would often be better supported throughout their childhood, and particularly during university. Participants referred to the detrimental effects that low socioeconomic status and financial stress can have on an individual’s mental health. These findings are not exclusive to this study, as it has been found that children and adolescents from families which are socioeconomically disadvantaged are more likely to experience poor mental wellbeing (Reiss, 2013). The findings from the current study and previous studies demonstrate the importance of providing additional support throughout school and university for those individuals from disadvantaged backgrounds. This could prove challenging as it is not money that has been shown to improve wellbeing, but the support that socioeconomic status enables families to provide.

Participants in the current study recognized that parents often bring their children up similarly to how they themselves were raised, and that support styles are passed down through generations. It was reported that the support individuals receive as children can have long lasting effects. Students emphasized that support provides a basis for adulthood, in particular contributing to healthy relationship formation later in life. This is supported by research showing that patterns of adult health are established during adolescence (Sawyer et al., 2012). Literature on the intergenerational transmission of parenting styles was absent until the recent publication of a longitudinal study spanning three generations (Abraham et al., 2021). The study investigated parenting styles, depression temperament and social support, finding evidence that the care individuals receive growing up can impact how they bond with their own children. Results showed that both adaptive and maladaptive parenting styles can be passed down, reflecting the opinions of participants in the current study. However, this is not to say that transmission of parenting styles is inevitable. Research has found that self-esteem may moderate the associations between parental depression and adolescent depression (Chang and Fu, 2020). If self-esteem can improve the wellbeing of individuals whose parents suffer with depression, perhaps other traits could mitigate the effects of poor parenting and reduce the likelihood of these behaviors being passed on. Future studies could focus on how the cycle of negative parenting styles can be broken, and how positive approaches to support can be encouraged.

In the current study, participants reported that support needs (including type and quantity) fluctuate across the lifespan. These findings mirror results obtained in previous studies which suggest that changes in independence lead to changes in the support required (van Harmelen et al., 2016). Students also expressed that support should remain available even in the absence of negative life events, which is consistent with existing literature on the topic. Turner and Brown (2010) outline a wealth of research for the importance of perceived support, emphasizing that “perceptions of support availability” are more important than the support received. Despite reporting that levels of required support alter throughout life, participants noted that support is important at any age, a finding which is reflected in the literature (Siedlecki et al., 2014). As previously mentioned, students in the current study believed support from parents to be the most essential. It would therefore be useful for future research to examine the perceived usefulness of different forms of social support during adulthood. This would allow for an exploration of whether parental support remains superior, or if this is replaced by support from friends and other peers.

Students in the current study stressed the importance of family support being adequate but not overbearing. Participants outlined how not having a support network with whom they can share their problems with can lead to these being exacerbated, along with how a lack of support can prompt individuals to seek help from other sources. Participants referenced divorce as a major cause of reduced parental support. This is consistent with research showing that negative family events can disrupt the support provided by parents, leading to negative consequences such as poor emotional wellbeing (Kroese et al., 2020). Findings also suggested that pressure and control can lead to friction within the parent-child relationship and reduce the benefits for wellbeing. Students reported that support was most helpful when it met their needs and it was neither overwhelming nor lacking. This is supported by research which suggests that, even when well-intentioned, excessive parental control can be detrimental (Grolnick, 2002). Researchers make a distinction between authoritative and authoritarian parenting (Haslam et al., 2020). Participants demonstrated an awareness of this difference, stating that authority figures (i.e., parents) who refrained from being controlling were essential for development. This study has contributed to the knowledge base surrounding family support, wellbeing and resilience, providing further evidence for the protective mechanism of social support (Armstrong et al., 2005). Results also built upon the existing literature, with findings demonstrating students to be particularly reliant on family support for maintaining their wellbeing whilst away at university. Findings from the current study suggest that resources aimed at educating support-givers on the differences between the two would be conducive to good wellbeing.

## 5 Limitations and future considerations

The current study explores the links between family support and wellbeing, addressing the lack of research into university students’ opinions of this topic. The study also aided in determining what students value most in terms of types of support. Although small, the sample contained individuals from several cultural backgrounds and thus a range of perspectives on family support were captured. However, with all 16 interviewees being from the same university and predominantly female, there are limits to sociodemographic diversity and diversity of perspectives, especially in terms of gender differences. Therefore, it cannot be assumed that these findings would be reflected in other populations. Furthermore, the current study included data from 15 females but only a single male. As a result, it is not possible to compare how understandings of family support may vary between these two groups, and findings may not fully represent the target demographic. Future research would benefit from a more heterogenous demographic from various institutions to capture a larger range of experiences and further enhance the applicability of findings. Future studies with more extensive and diverse samples might wish to further explore the risk factors associated with family support and poor wellbeing. This could help researchers gain a deeper understanding of how the effects of undesirable support can be mitigated, and aid in the development of interventions for those individuals from adverse family backgrounds. Lastly, considering the data collection method of semi-structured interviews, although rich and insightful, this method may not be consistent with the more objective quantitative findings in the field.

## 6 Conclusion

Wellbeing is inextricably linked with our quality of life, and there are many factors which can impact upon an individual’s experience of wellbeing (Deiner et al., 2009). Social support has been identified as integral for maintaining wellbeing, with many individuals viewing family support as being the most important aspect (Smart et al., 2008; Turner and Brown, 2010). Due to the developmental changes taking place in young people aged 10–19 years old, wellbeing during adolescence through to the university years has been a topic of interest for psychologists (Compas, 1987; Storrie et al., 2010). The current study explored the views of university students around family support and its link to wellbeing. It sought to address a gap in the literature and develop the knowledge available surrounding both the adverse and protective factors linked to family support and wellbeing throughout life. Findings revealed that university students valued the ability to talk about their problems with family members, often intending not to solve but simply to share them. Students also recognized the role that family context (such as cultural background and socioeconomic status) has in the support that is provided by a family. Participants regularly referred to the importance of support being available throughout all stages of life, and how it is necessary for this to be balanced without becoming oppressive.

Given the findings, future research could investigate what university students understand about maintaining their own wellbeing. Although family support is clearly influential, research has found that how an individual copes with stress is often more impactful for wellbeing than the stressful event itself (Lohman and Jarvis, 2000). This suggests that family support contributes to the development of coping mechanisms. Thus, gaining a greater understanding of how to improve the coping strategies of university students may be the next step in helping those who have not received adequate support throughout childhood or adolescence. As university is a distressing time of life (Storrie et al., 2010), more funding should be allocated so that support services are readily available for students that don’t have access to good family support. Research should then extend to understand how services can support adults at risk of poor wellbeing due to family circumstances earlier on in life.

## Data Availability

The raw data supporting the conclusions of this article will be made available by the authors on request from the corresponding author, without undue reservation.

## Appendix A

**APPENDIX TABLE 1:** Participants’ ethnicity information.

Ethnicity	Total
Asian	1
Asian-Indian	1
British Pakistani	1
British Turkish	1
Chinese	2
Pakistani	1
White	2
White British	5
Indian	1
N/A	1

## Appendix B

Topic Guide

1. General family support

– What does family support mean to you?
– How do you understand family support?
– How important do you think this is growing up for individuals?
– What impact do you think it can have on individuals growing up?

2. Family context/cultural/financial background

– Do you think an individual’s cultural background could impact the type/quality of family support they receive? In what way? Why?
– Do you think an individual’s socio-economical background could impact the family support they receive? In what way? Why?
– Is there anything else that you think could impact the family support for individuals growing up?
– What do you think family support should look like? Are there any specific areas of family support that are more important than others? Why? In what why? What is the impact of this?

3. Wellbeing

– Do you think family support can impact an individual’s wellbeing? In what way? Can that impact change over time? In what way?
– Do you think family support impacts an individual’s ability to deal with everyday stressors and challenging situations? In what way?
– What aspects of family support do you think could positively impact wellbeing? How? Why?
– What aspects of family support do you think could negatively impact wellbeing? How? Why?

Any other comments related to this topic?

Thank you for taking part in the study. I hope this has not been upsetting for you in any way.

Share debrief form.
